# Aromatase deficiency in a tall man: Case report of two novel mutations and review of literature

**DOI:** 10.1016/j.bonr.2022.101642

**Published:** 2022-11-23

**Authors:** Pankaj Singhania, Debasish Dash, Abhranil Dhar, Pritam Biswas, Piyas Gargari, Rana Bhattacharjee, Subhankar Chowdhury, Dipanjana Datta, Emili Banerjee

**Affiliations:** a Department of Endocrinology and Metabolism, Institute of Post Graduate Medical Education and Research/SSKM Hospital, 244, AJC Bose Road, Kolkata 700020, West Bengal, India; b Institute of Child Health, Kolkata, India; c Lifecell International Pvt Ltd, India; d Organization of Rare Disease, Bengaluru, India

**Keywords:** LH, luteinising hormone, FSH, follicle stimulating hormone, IGF1, insulin like growth factor 1, DXA, dual energy X-ray absorptiometry, Aromatase, Knock knee, Tall stature, Estradiol, Epiphysis

## Abstract

Aromatase (CYP19A1) is the only enzyme known to catalyse the conversion of androgen to estrogen. Aromatase deficiency occurs due to mutation in *CYP19A1*gene which has an autosomal recessive inheritance pattern. It leads to decrease in estrogen synthesis and delayed epiphyseal closure, eunuchoid habitus and osteopenia. We are presenting here, a 24 years old male, with history of progressive increase in height and knock knees. X-ray showed open wrist and knee epiphysis. The serum testosterone level was normal and serum estradiol level was undetectable. Semen analysis showed azoospermia. Clinical exome sequencing gave two novel mutations in *CYP19A1*. The first variant was a novel single nucleotide deletion of thiamine at 570th base of the cDNA (c.570delT) of *CYP19A1* gene. The second variant detected was again a novel one in the same gene in Exon 5 corresponding 344th base of the cDNA (c344G>A) resulting in a missense mutation of 115th arginine to glutamine in the protein. Sanger sequencing showed that the later mutation was inherited from the father. The patient was started on oral estradiol valerate for epiphyseal closure to prevent further increase in height. Only 15 mutations have been reported in the aromatase gene in males till date, our report of these novel mutations will be an add-on to the literature.

## 1 Introduction

Aromatase is a member of the cytochrome P450 family and a product of the *CYP19A1* gene (Simpson, 2004). It is the rate-limiting enzyme in the conversion of androstenedione to estrone (E1) and testosterone to estradiol (E2) and is critical for the production of estrogen in both sexes (Ghosh et al., 2009; Milani et al., 2009). Aromatase is expressed heavily in the human placenta and ovarian follicles. It is also expressed at a number of other sites like Leydig and Sertoli cells of the testes, breast, neurons, brain, liver, pre adipocytes, fibroblast, chondrocytes, and osteoblasts (Simpson, 2003). Aromatase deficiency is an autosomal recessive disorder due to mutation of the *CYP19A1* gene which presents in females with pseudo hermaphroditism and males with tall stature, delayed epiphyseal closure, and osteopenia due to impaired bone mineralization (Carani et al., 1997). The study of aromatase-deficient males confirms the critical role of estrogen in males, particularly in spermatogenesis and bone maturation (Morishima et al., 1995).

## 2 The case

**Fig. 1:**
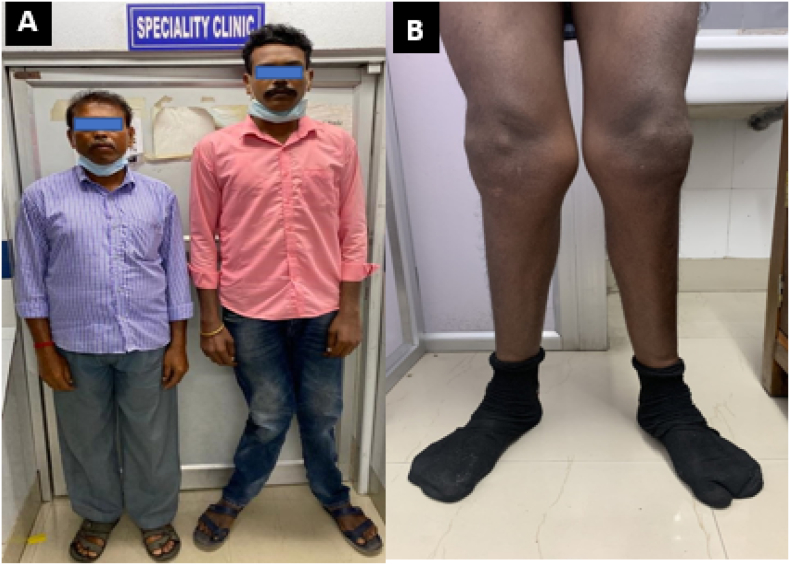
The patient height compared to his father showing tall stature and knock knees (A). Closer view of the legs of the patient showing knock knees or genu valgum (B).

**Fig. 2:**
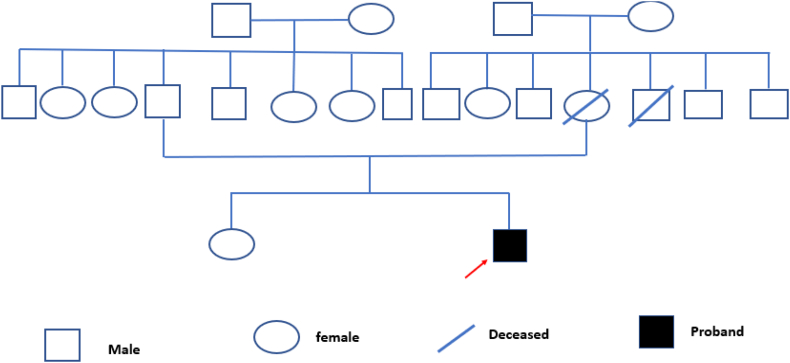
Pedigree chart of the family.

A 24-year-old male presented with progressive increase in stature (Fig. 1A) even after 18 years of age such that he grew taller than his peers and he was the tallest in his family. As he grew tall, he also developed bilateral knock knees and difficulty in walking. He had not sustained any fracture in his lifetime. He had normal libido and was yet to father a child. There is no such illness in the family (Fig. 2). His father was 170 cm tall. His mother died of breast cancer and his elder sister is married with one child. He was 188 cm tall with an arm span of 200 cm.The upper segment lower segment ratio was 0.8. There was prominent outward bending of both legs at the knee suggestive of genu valgum with an inter-malleolar distance of 24 cm (Fig. 1B) and Q Angle = Right 28^0,^ Left 26^0.^

**Fig. 3:**
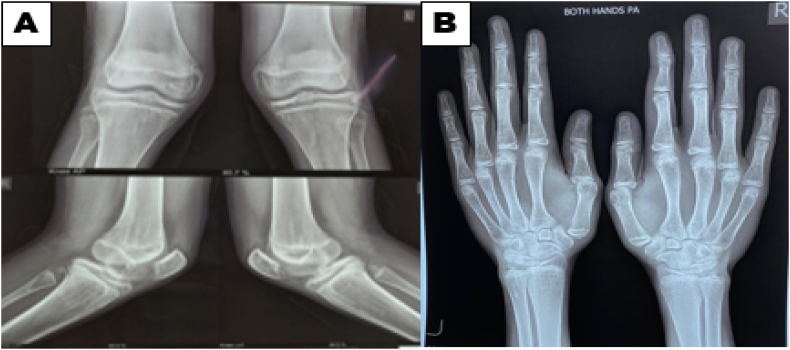
X- ray knee joints (A) and wrist (B) showing open epiphysis.

His sexual maturity rating was tanner 5 and he had adequate facial hair. Skiagrams of knee joint and wrist joints showed that the epiphysis was unfused (Fig. 3).

Biochemical investigations obtained are shown in Table 1. He was euthyroid, had normal blood glucose levels with no features of insulin resistance and his lipid profile was essentially normal. Bone mineral density (BMD) determined by DXA scan gave a Z score of −2.5 at both neck femur and −2.6 at the lumbar spine. The corresponding T-score was −2.5 and −2.6 respectively. Semen analysis showed azoospermia. Clinical exome sequencing for suspected aromatase deficiency and *CYP19A1* mutation was undertaken. The first variant detected was a novel single nucleotide deletion of Thiamine at the 570th base of the cDNA (c.570delT) of the *CYP19A1* gene. This deletion resulted in an alteration of Leucine 191 to Cysteine 191 and a reading frameshift of ribosomes so that the following 14th codon was converted to a stop codon. The protein synthesized by *CYP19A1* is aromatase having 503 amino acids. In our patient, this frameshift mutation resulted in a truncated non-functional aromatase protein of 204 amino acids. According to gnomAD and 1 kG databases, this region (where this variation has occurred) of the gene has been reported for at least 14 other pathogenic variations. Therefore, the amino acid sequence of this region is critical for protein functioning and this novel frameshift mutation has altered the amino acid composition leading to a non-functional truncated protein This mutation was reported as likely pathogenic according to the American College of Medical Genetics and Genomics (ACMG) classification.

**Table 1:** Baseline and follow up biochemistry of the patient.

Parameter	Reference value	Baseline	3 months after treatment
Serum estradiol (pg/ml)	10–82	<20	25.4
Serum testosterone (mg/dl)	350–1080	626	712
Serum sex hormone binding globulin (SHBG) nmol/L	18.3–54.1	28.47	–
Serum FSH (U/L)	1–8	12.2	8.96
Serum LH (U/L)	1.8–12	5.18	3.81
Serum IGF1 (ng/ml)	114–492	172	–
Total cholesterol (mg/dl)	<200	210	202
LDL (mg/dl)	<130	132	83.2
HDL (mg/dl)	>55	42	67.3
Serum triglyceride (mg/dl)	<150	121	260
Fasting glucose (mg/dl)	60–100	96	73
Serum ALP (U/L)	40–129	130	244

The second variant detected was again a novel one in the same gene in Exon 5 corresponding to the 344th base of the cDNA (c344G>A) resulting in a missense mutation of the 115th arginine to glutamine in the protein. The amino acid arginine at the 115th position of *CYP19A1* protein is conserved throughout the mammalian world as was evident from GERP++ and PHYLoP databases. SIFT and Polyphen2 databases have predicted the change of Arg115Gln as damaging. This was a variant of uncertain significance as per ACMG classification.

To access the effect of the R115Q mutation on function of the protein, docking of the ligand (PROTOPORPHYRIN IX CONTAINING FE) was carried out using Autodock for both wild and mutant. The mutant version has high binding energy (−14.89) when compared with wild version (−19.39), which indicates that mutations have reduced the binding affinity, thereby reducing its efficacy with the substrate. In order to evaluate the structural changes due to mutation at active site the comparison of the interacting amino acid of wild & mutant ligand and protein complex was done using plip. The wild *CYP19A1* protein interacts with ligand (PROTOPORPHYRIN IX CONTAINING FE) with the help of 3 hydrogen bonds, 7 hydrophobic interactions and 5 salt bridge interactions while mutant protein utilizes only 1 hydrogen bond, 9 hydrophobic interactions and 3 salt bridge interactions. The loss of hydrogen bond and salt bridges interaction in mutant resulted in reduced binding affinity with the substrate, thereby affecting the functional efficacy of the protein. The results are tabulated. The CoNSURF analysis also indicated residues R115 belongs to highly conserved region. The results are summarized in the Table 2 and pictures are given as a supplementary file (supplementary file 1).

**Table 2:** Summary of the functional study through computational modelling.

S. no	AA substitution	I-Mutant	HOPE Server	Docking score	Evolutionary conservation	Protein domain
1	Arg115Gln (R115Q)	Decrease	Deleterious (Difference in charge & size of the amino acid Mutant amino acid is smaller Mutant amino acid is Neutral while Wild is positively charged Occurs in highly conserved region)	Wild: −19.39 Mutant: −14.89	Wild: Highly conserved & buried	Cytochrome P450 family 19
2	Leu191Cysfs*14	Disease causing	Loss of a large chunk of secondary structure elements	N/A	N/A	

**Fig. 4:**
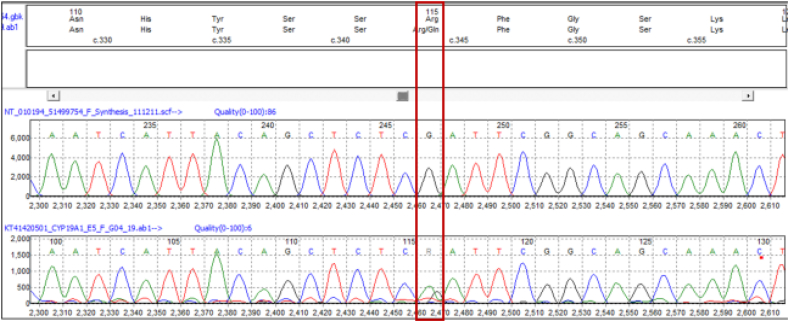
Sanger sequencing data (electropherogram) for the father showing nucleotide change at chr15: c.344G>A, (p. Arg115Gln) in *CYP19A1* gene which was detected in the proband.

Sanger sequencing of the parents was planned for mutation confirmation. Since only his father was alive, Sanger sequencing of the father was undertaken which showed the presence of the later mutation (chr 15: c.344G>A, p. Arg115Gln) in heterozygous form (Fig. 4).

The heritability of one of the detected mutations from the father further strengthened our diagnosis.

The patient was started on tablet estradiol valerate 0.5 mg once daily to facilitate epiphyseal fusion and halt height progression. Biochemical analysis 3 months after therapy is shown in Table 1. Results show an increase in serum estradiol, and HDL and a decrease in levels of gonadotrophins. Though there is an increase in triglyceride level, he remains euglycemic. Correction of genu valgum and fertility issues is discussed and will be planned at a later point of time after epiphyseal closure.

## 3 Discussion

First described in 1995, aromatase deficiency due to aromatase mutation is an autosomal recessive disorder associated with impaired estradiol synthesis. The implicated mutation is in the *CYP19A1* gene. Estrogen, as a female hormone has long been recognised due to its profound importance in women of reproductive age group. The role of estrogen in men and the non-reproductive effects of estrogen in women are also important particularly in the context of bone health and maturation (Deroo and Korach, 2006).

At puberty, growth and bone maturation depend on androgen in both genders. Androgen turns into estrogen by aromatase in men. Estrogen levels increase due to increasing androgens at puberty and lead to bone maturation and ossification at the epiphyseal cartilages (Klein et al., 1996). In the absence of aromatase there is delayed epiphyseal closure leading to tall stature as in our case. Additional findings in men include genu valgum or knock knees, eunuchoid body proportions, and osteopenia or osteoporosis. Impaired fertility, low libido and cryptorchidism have also been reported. Most of these manifestations are present in the index case (Deladoëy et al., 1999; Maffei et al., 2004; Mittre-Hervé et al., 2004; Lin et al., 2007; Maffei et al., 2007; Lanfranco et al., 2008; Jones et al., 2006).

Similar to our case, hormonal analysis in men with aromatase deficiency generally show elevated gonadotrophins with normal testosterone and low or undetectable estradiol levels. This proves that estrogen regulates LH and FSH in men by a negative feedback loop (Rochira et al., 2006).

Aromatase enzyme activity and its absence become evident in men by late puberty and men with aromatase deficiency are normal before puberty. The development of our patient was also normal until puberty. His linear growth continued after puberty so that he got taller than the target height of his family.

Genetic analysis of the *CYP19A1* mutation is key to making a definitive diagnosis of aromatase deficiency. The mutations reported here within the *CYP19A1* gene lies in the substrate binding domain of the protein. Therefore, any change in this region would seriously be affecting the function of the protein. The circulating testosterone level of 626 ng/dL and undetectable estradiol level are a result of this compromised aromatase activity. Mutation identified in our patient is compound heterozygous condition as understood from the segregation studies.

Till date there are 15 reports of mutations in *CYP19A1* reported in males. The clinical, biochemical and genetic details of these cases are shown in Table 3. Similar to all previous reported cases, our case was also tall and has a wide arm span. The cases were also similar in having high testosterone, low estradiol, raised gonadotrophins and low bone mineral density. Our case unlike most previous reported cases had no history of low trauma fracture. Azoospermia was also present in our case unlike most other reported cases.

**Table 3:** Details of reported aromatase deficiency cases in males.

Sl no.	Author	Mutation reported	Phenotype	Journal
1	Susanne U Miedlich et al.	Homozygous c.628G>A, exon 5, CYP19A1	Young male, tall, pathologic fracture, unfused epiphyses, low bone mass	Miedlich SU, Karamooz N, Hammes SR. Aromatase deficiency in a male patient - Case report and review of the literature. Bone. 2016 Dec;93:181–186. doi: 10.1016/j.bone.2016.09.024. Epub 2016 Sep 29. PMID: 27693882.
2	Hongli Li et al.	Homozygous c.1093C>T, p.R365W, CYP19A1	Young adult male, delayed bone age, unfused epiphyses, low bone mass, low Estradiol, high gonadotrophin	Li H, Fu S, Dai R, Sheng Z, Liu W. Aromatase deficiency caused by mutation of CYP19A1 gene: A case report. Zhong Nan Da Xue Xue Bao Yi Xue Ban. 2022 Jun 28;47(6):794–800. English, Chinese. doi: 10.11817/j.issn.1672-7347.2022.210401. PMID: 35837780.
3	Emine Kartal Baykan et al.	Homozygous R375H G-A	Bone pain, recurrent pathologic fractures, tall stature, enunchoid, osteoporosis	Baykan EK, Erdoğan M, Özen S, Darcan Ş, Saygılı LF. Aromatase deficiency, a rare syndrome: case report. J Clin Res Pediatr Endocrinol. 2013;5(2):129–32. doi: 10.4274/Jcrpe.970. Erratum in: J Clin Res Pediatr Endocrinol. 2013 Sep 10;5(3):216. PMID: 23748068; PMCID: PMC3701920.
4	A Morishima et al.	Homozygous, c1123C>T, Exon 9. p. R375C	Male, tall stature, enunchoid, delayed skeletal age, osteoporosis, high gonadotrophins, high gonadal steroids.	Morishima A, Grumbach MM, Simpson ER, Fisher C, Qin K. Aromatase deficiency in male and female siblings caused by a novel mutation and the physiological role of estrogens. J Clin Endocrinol Metab. 1995 Dec;80(12):3689–98. doi: 10.1210/jcem.80.12.8530621. PMID: 8530621.
5	Mariana Costanzo et al.	Homozygous mutation (Arg192Cys) in the CYP19A1	Isosexual precosity, tall stature, osteopenia.	Costanzo M, Garcia-Feyling J, Saraco N, Marino R, Pérez Garrido N, Touzon MS, Viterbo G, Lazzati JM, Patiño HC, Mattone C, Maceiras M, Belgorosky A, Guercio G. Accelerated Pubertal Tempo in a 46,XY Aromatase-Deficient Patient. Horm Res Paediatr. 2018;90(4):275–282. doi: 10.1159/000492128. Epub 2018 Aug 31. PMID: 30173221.
6	B L Herrmann et al.	Homozygous, CYP19A1	Tall stature, enunchoid, genu valgum, khyphoscoliosis, abdominal striae, pectus carinatum, low bone mass	Herrmann BL, Janssen OE, Hahn S, Broecker-Preuss M, Mann K. Effects of estrogen replacement therapy on bone and glucose metabolism in a male with congenital aromatase deficiency. Horm Metab Res. 2005 Mar;37(3):178–83. doi: 10.1055/s-2005-861292. PMID: 15824973.
7	Zhike Chen, et al.	Compound heterozygous CYP19A1 mutations (Y81C and L451P)	Tall stature, enunchoid, genu valgum, unfused epiphyses, osteopenia, Metabolic syndrome.	Chen Z, Wang O, Nie M, Elison K, Zhou D, Li M, Jiang Y, Xia W, Meng X, Chen S, Xing X. Aromatase deficiency in a Chinese adult man caused by novel compound heterozygous CYP19A1 mutations: effects of estrogen replacement therapy on the bone, lipid, liver and glucose metabolism. Mol Cell Endocrinol. 2015 Jan 5;399:32–42. doi: 10.1016/j.mce.2014.09.016. Epub 2014 Oct 6. PMID: 25301327; PMCID: PMC4457386.
8	Laura Maffei et al.	Inactivating mutation, 1 bp (c) deletion, exon V, CYP19A1 (Done by PCR, genome seq not done)	Persistent linear growth, bone pain, cryptorchidism, enunchoid, genu valgum, osteoporosis	Maffei L, Murata Y, Rochira V, Tubert G, Aranda C, Vazquez M, Clyne CD, Maffei L, Murata Y, Rochira V, Tubert G, Aranda C, Vazquez M, Clyne CD, Davis S, Simpson ER, Carani C. Dysmetabolic syndrome in a man with a novel mutation of the aromatase gene: effects of testosterone, alendronate, and estradiol treatment. J Clin Endocrinol Metab. 2004 Jan;89(1):61–70. doi: 10.1210/jc.2003-030313. PMID: 14715828.
9	Deladoey et al	Homozygous for a C-base deletion which causes a frameshift mutation and produces a stop codon after 21 codons	Hyperadrogenism in mother; very low estradiol, normal testosterone, and normal gonadotrophins in child.	Deladoëy J, Flück C, Bex M, Yoshimura N, Harada N, Mullis PE. Aromatase deDeladoëy J, Flück C, Bex M, Yoshimura N, Harada N, Mullis PE. Aromatase deficiency caused by a novel P450arom gene mutation: impact of absent estrogen production on serum gonadotropin concentration in a boy. J Clin Endocrinol Metab. 1999 Nov;84(11):4050–4. doi: 10.1210/jcem.84.11.6135. PMID: 10566648.
10	Fabio Lanfranco et al.	Compound heterozygosity due to 23 bp deletion in exon IV and a point mutation in the first nucleotide of intron IX of the CYP19A1 gene	Progressive tall stature, Obese, unfused epiphyses, undetectable estradiol.	Lanfranco F, Zirilli L, Baldi M, Pignatti E, Corneli G, Ghigo E, Aimaretti G, Carani C, Rochira V. A novel mutation in the human aromatase gene: insights on the relationship among serum estradiol, longitudinal growth and bone mineral density in an adult man under estrogen replacement treatment. Bone. 2008 Sep;43(3):628–35. doi: 10.1016/j.bone.2008.05.011. Epub 2008 May 23. PMID: 18590994.
11	C Carani et al.	Single G → A mutation at base pair (bp) 1094 in exon 9, P-450 aromatase gene.This mutation abolishes a site cleaved by the restriction enzyme Acc651,	Male, progressive height gain, unfused epiphyses, osteopenia, genu valgum, undetectable estradiol, normal gonadotrophins.	Carani C, Qin K, Simoni M, Faustini-Fustini M, SerpCarani C, Qin K, Simoni M, Faustini-Fustini M, Serpente S, Boyd J, Korach KS, Simpson ER. Effect of testosterone and estradiol in a man with aromatase deficiency. N Engl J Med. 1997 Jul 10;337(2):91–5. doi: 10.1056/NEJM199707103370204. PMID: 9211678.
12	Laura Maffei et al.	Compund heterozygous mutation, bp380 (T→G) in exon IV and the second one at bp 1124 (G→A) in exon IX.	Tall stature, enunchoid, diffuse bone pain, central obesity, insulin resistance, NAFLD, low estradiol level.	Maffei L, Rochira V, Zirilli L, Antunez P, Aranda C, Fabre Maffei L, Rochira V, Zirilli L, Antunez P, Aranda C, Fabre B, Simone ML, Pignatti E, Simpson ER, Houssami S, Clyne CD, Carani C. A novel compound heterozygous mutation of the aromatase gene in an adult man: reinforced evidence on the relationship between congenital estrogen deficiency, adiposity and the metabolic syndrome. Clin Endocrinol (Oxf). 2007 Aug;67(2):218–24. doi: 10.1111/j.1365-2265.2007.02864.x. Epub 2007 Jun 4. PMID: 17547681.
14	Sun Young Kim et al	Homozygous variant in CYP19A1 gene c.1304G>A (p.Arg435His)	Not available	Kim SY, Colaiacovo S, Dave S, Coughlin K, Langdon K, Stein R, Saleh M. AromatKim SY, Colaiacovo S, Dave S, Coughlin K, Langdon K, Stein R, Saleh M. Aromatase deficiency in an Ontario Old Order Mennonite family. J Pediatr Endocrinol Metab. 2021 Aug 5;34(12):1615–1618. doi: 10.1515/jpem-2021-0229. PMID: 34348419.
15	Bouillion R et al	Homozygous for a C-base deletion which causes a frameshift mutation and produces a stop codon after 21 codons	Male, obese, congenital deafness, open epiphyses, low bone mass, low esradiol and normal gonadotrophins.	Bouillon R, Bex M, Vanderschueren D, Boonen S. Estrogens are essential for male pubertal periosteal bone expansion. J Clin Endocrinol Metab. 2004 Dec;89(12):6025–9. doi: 10.1210/jc.2004-0602. PMID: 15579754.

Estradiol is the final product of aromatase enzyme and so estrogen replacement is the mainstay of therapy in aromatase deficiency. Bone maturation and epiphyseal closure can be accelerated with estrogen therapy. This will stop further increase in height and residual deformities have to be corrected surgically. High dose estrogen (25–50 μg/day) is used in these cases (Rochira et al., 2002).

### 3.1 Limitation

Follow up data is yet to be compiled in our case. Follow up for epiphyseal closure and azoospermia after 1–2 years may add more information to the presentation. Growth curve for the subject was not available from medical records, which could have given the timeline for increase in height.

## CRediT authorship contribution statement

Dr. Pankaj Singhania conceptualised the case and assimilated all the information and made final editing. Dr. Debasish Dash, Dr. Abhranil Dhar, Dr. Pritam Biswas, and Dr. Piyas Gargari helped to prepare the initial draft and were involved in care of the patient. Dr. Rana Bhattacharjee and Dr. Subhankar Chowdhury supervised the whole venture, oversaw the writings and gave expert opinion. Dr. Emili Banerjee and Dr. Dipanjana Datta derived genetic conclusions and gave valuable genetic inputs.

## Written informed consent

Obtained.

## Declaration of competing interest

None.

## Data Availability

Data will be made available on request.

## Appendix A Supplementary data

Supplementary material Image 1
