# Supplementary material for: Aromatase deficiency in a tall man: Case report of two novel mutations and review of literature
Source: Bone Rep. 2022 Nov 23;17:101642. doi: 10.1016/j.bonr.2022.101642 (PMC9732115; doi:10.1016/j.bonr.2022.101642)
Supplement: Supplementary file 1 — Supplementary material [file mmc1.pdf]

All figures

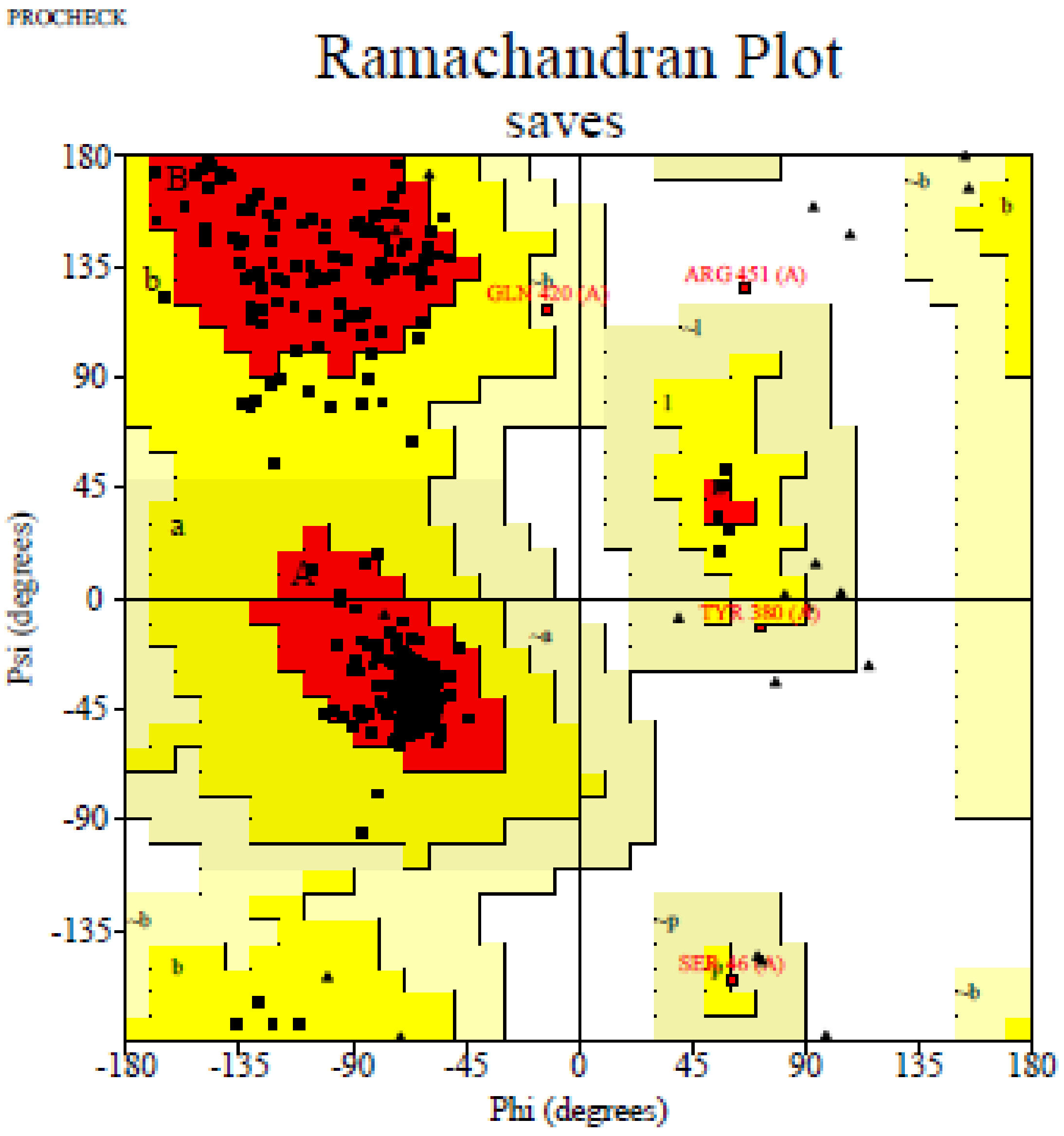

Plot statistics

Plot statistics

Residues in most favoured regions [A,B,L]	371	91.8%
Residues in additional allowed regions [a,b,l,p]	29	7.2%
Residues in generously allowed regions [~a,~b,~l,~p]	3	0.7%
Residues in disallowed regions	1	0.2%
	----	------
Number of non-glycine and non-proline residues	404	100.0%
Number of end-residues (excl. Gly and Pro)	6	
Number of glycine residues (shown as triangles)	27	
Number of proline residues	19	

Total number of residues	456	

Procheck Result

```
*************************************************************************
**                                                                     **
**                          I-Mutant v2.0                              **
**         Predictor of Protein Stability Changes upon Mutations       **
**                                                                     **
*************************************************************************


      PDB File: pdb3eqm.ent    Chain: A

      Position   WT  NEW   Stability  RI    pH    T   RSA
           115    R    Q    Decrease   8   7.0   25  16.6


      WT:  Aminoacid in Wild-Type Protein
      NEW: New Aminoacid after Mutation
      RI:  Reliability Index
      T:   Temperature in Celsius degrees
      pH:  -log[H+]
      RSA: Relative Solvent Accessible Area



*************************************************************************
*                                                                       *
* Capriotti E, Fariselli P and Casadio R (2005). I-Mutant2.0: predicting *
* stability changes upon mutation from the protein sequence or structure. *
* Nucl. Acids Res. 33: W306-W310.                                       *
* http://gpcr.biocomp.unibo.it/cgi/predictors/I-Mutant2.0/I-Mutant2.0.cgi *
*                                                                       *
*************************************************************************
```

I Mutant Analysis:
Shows Mutation decreases the stability of the protein.

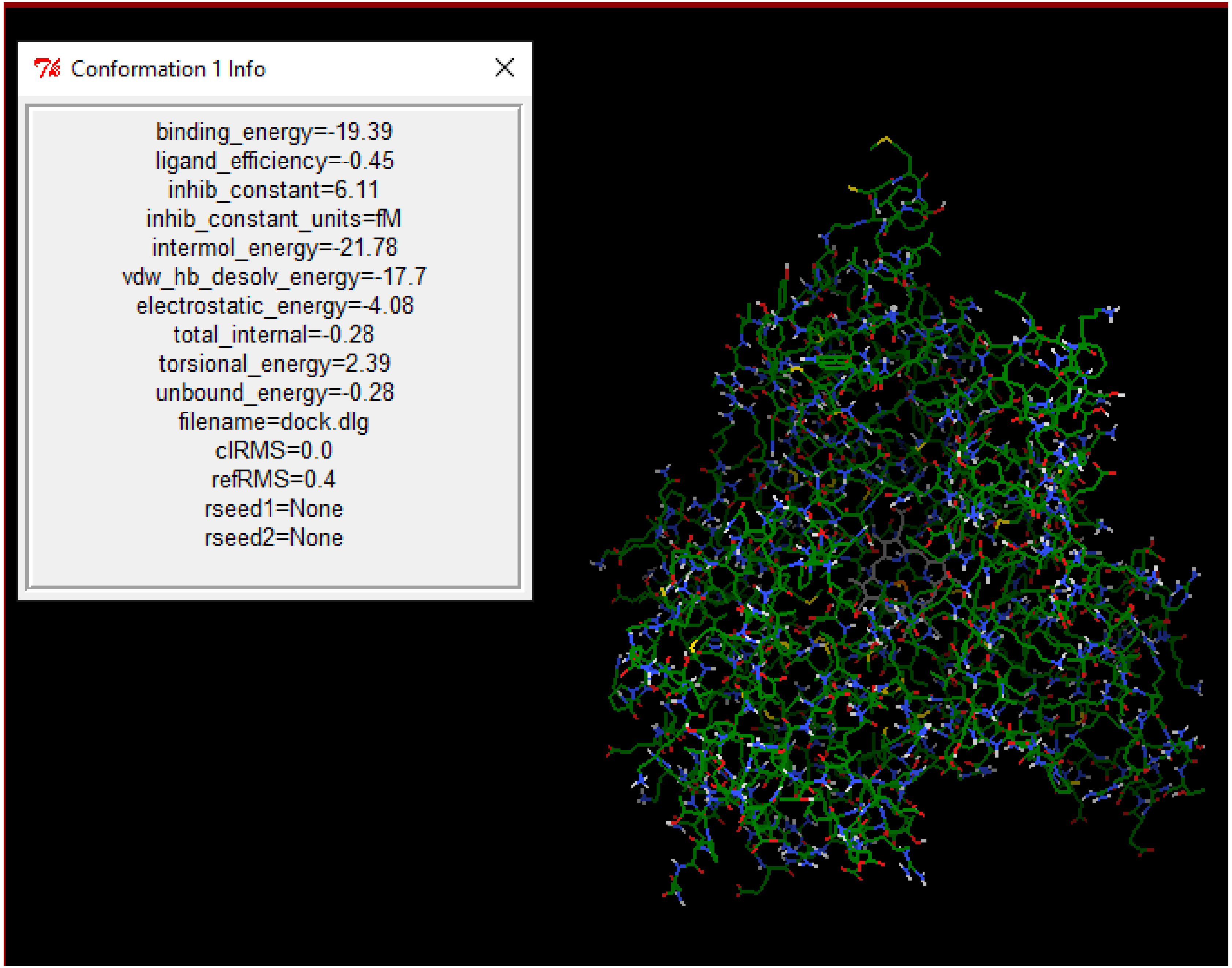

Autodock Result
Wild

Hydrophobic Interactions

Index	Residue	AA	Distance	Ligand Atom	Protein Atom
1	152A	LEU	3.71	4507	1057
2	306A	ALA	3.67	4508	2578
3	370A	VAL	3.93	4480	3207
4	370A	VAL	3.70	4474	3206
5	373A	VAL	3.73	4474	3233
6	430A	PHE	3.46	4478	3820
7	438A	ALA	3.73	4490	3885

Hydrogen Bonds

Index	Residue	AA	Distance H-A	Distance D-A	Donor Angle	Protein donor?	Side chain	Donor Atom	Acceptor Atom
1	133A	ILE	3.24	3.63	107.65	✗	✗	4503 [O.co2]	850 [O2]
2	141A	TRP	1.99	3.01	177.60	✓	✓	935 [Nar]	4504 [O.co2]
3	435A	ARG	2.26	2.71	108.54	✗	✗	4499 [O.co2]	3855 [O2]

Plip Protein ligand interaction result Result Wild

Salt Bridges

Index	Residue	AA	Distance	Protein positive?	Ligand Group	Ligand Atoms
1	115A	ARG	4.36	✓	Carboxylate	4499, 4500
2	115A	ARG	4.65	✓	Carboxylate	4503, 4504
3	145A	ARG	4.79	✓	Carboxylate	4503, 4504
4	375A	ARG	3.59	✓	Carboxylate	4499, 4500
5	435A	ARG	3.92	✓	Carboxylate	4503, 4504

Plip Protein ligand interaction result Result Wild

Metal Complexes

Index	Residue	AA	Metal	Target	Distance	Location
Complex 1: Fe, square.pyramidal (5)						
1	437A	CYS	4496	3879	2.20	protein.sidechain
2	600A	HEM	4496	4492	2.04	ligand
3	600A	HEM	4496	4493	2.02	ligand
4	600A	HEM	4496	4494	2.01	ligand
5	600A	HEM	4496	4495	2.09	ligand

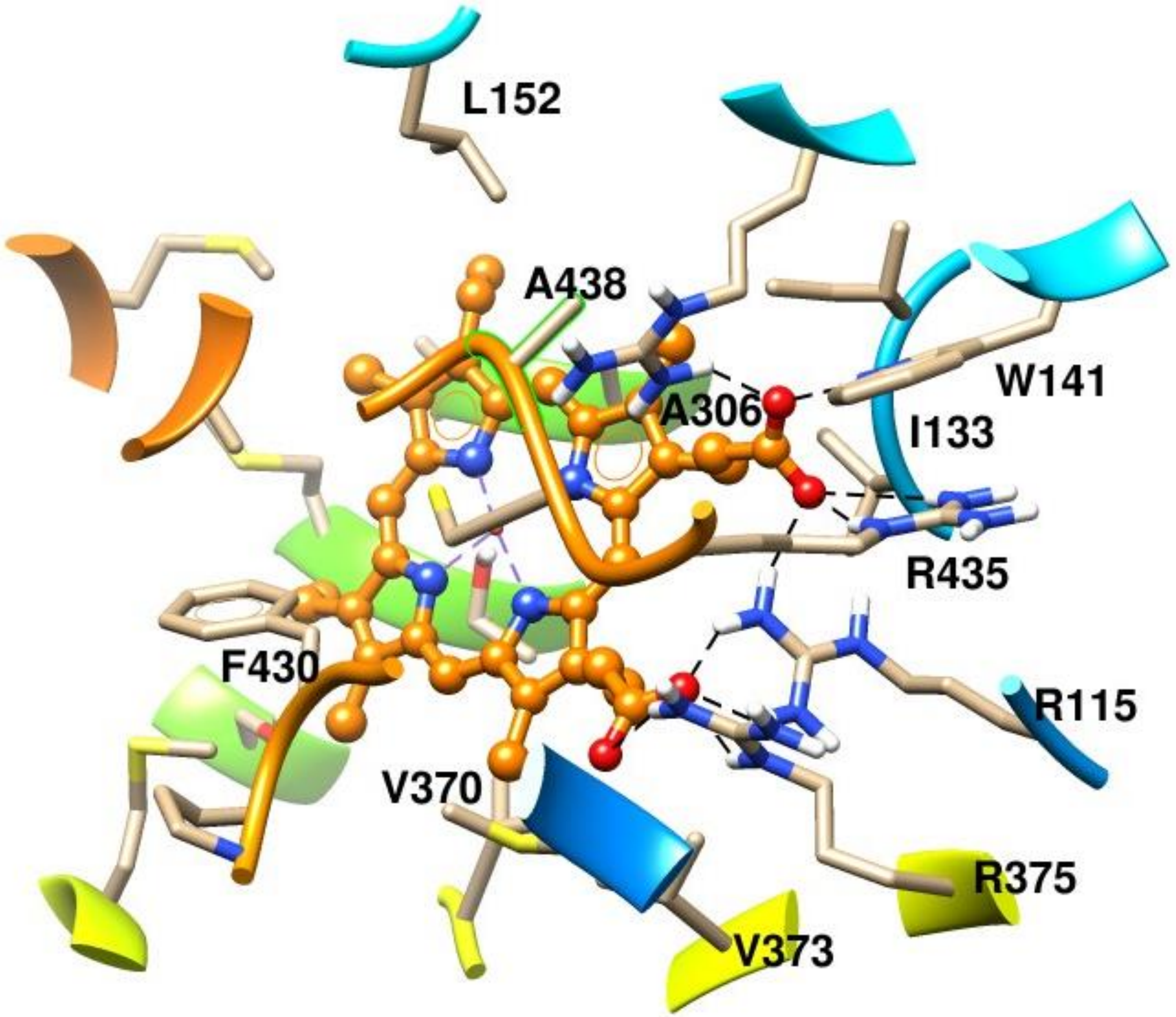

Protein ligand interaction result
Result Wild

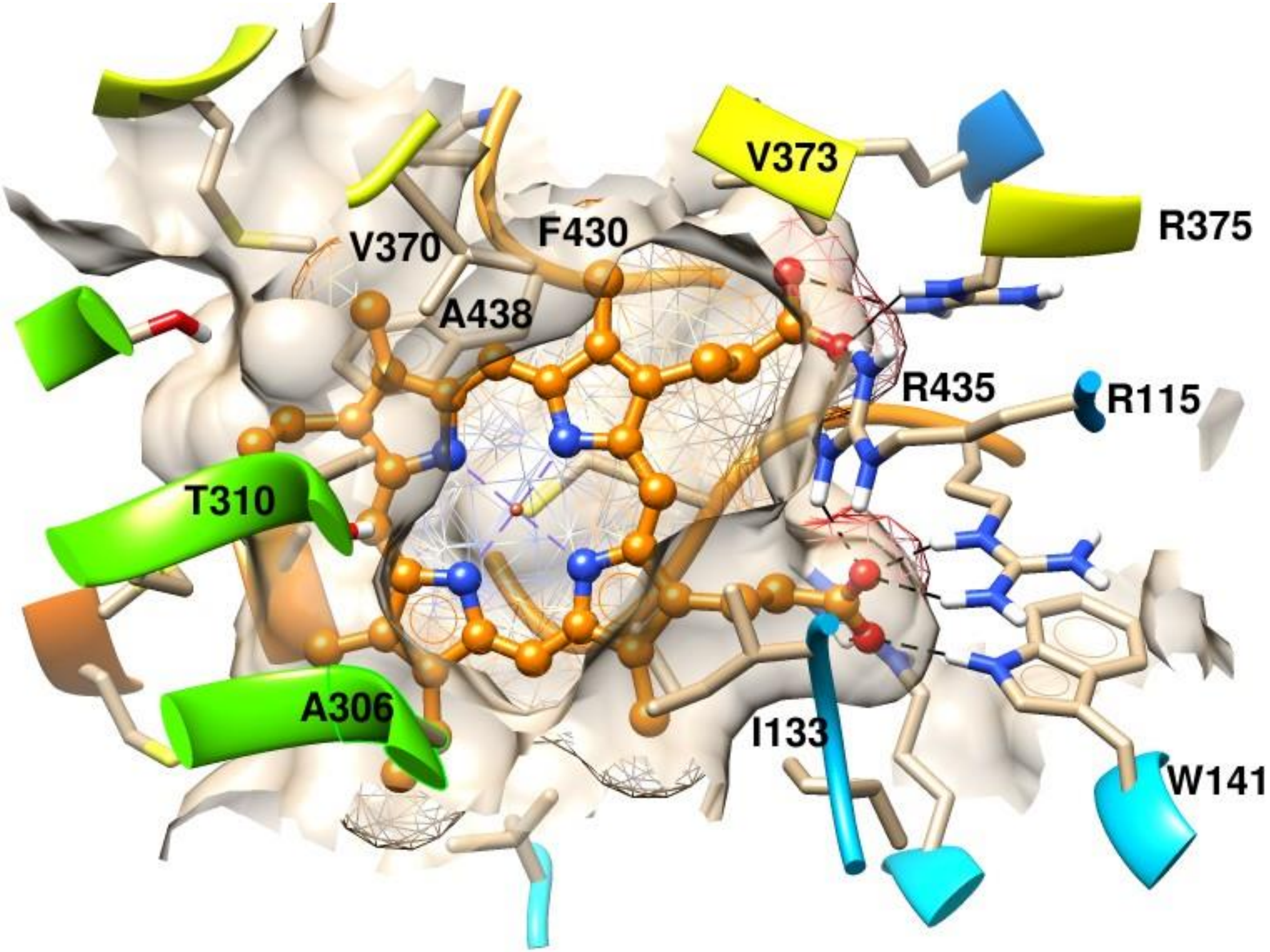

Protein ligand interaction result
Result Wild

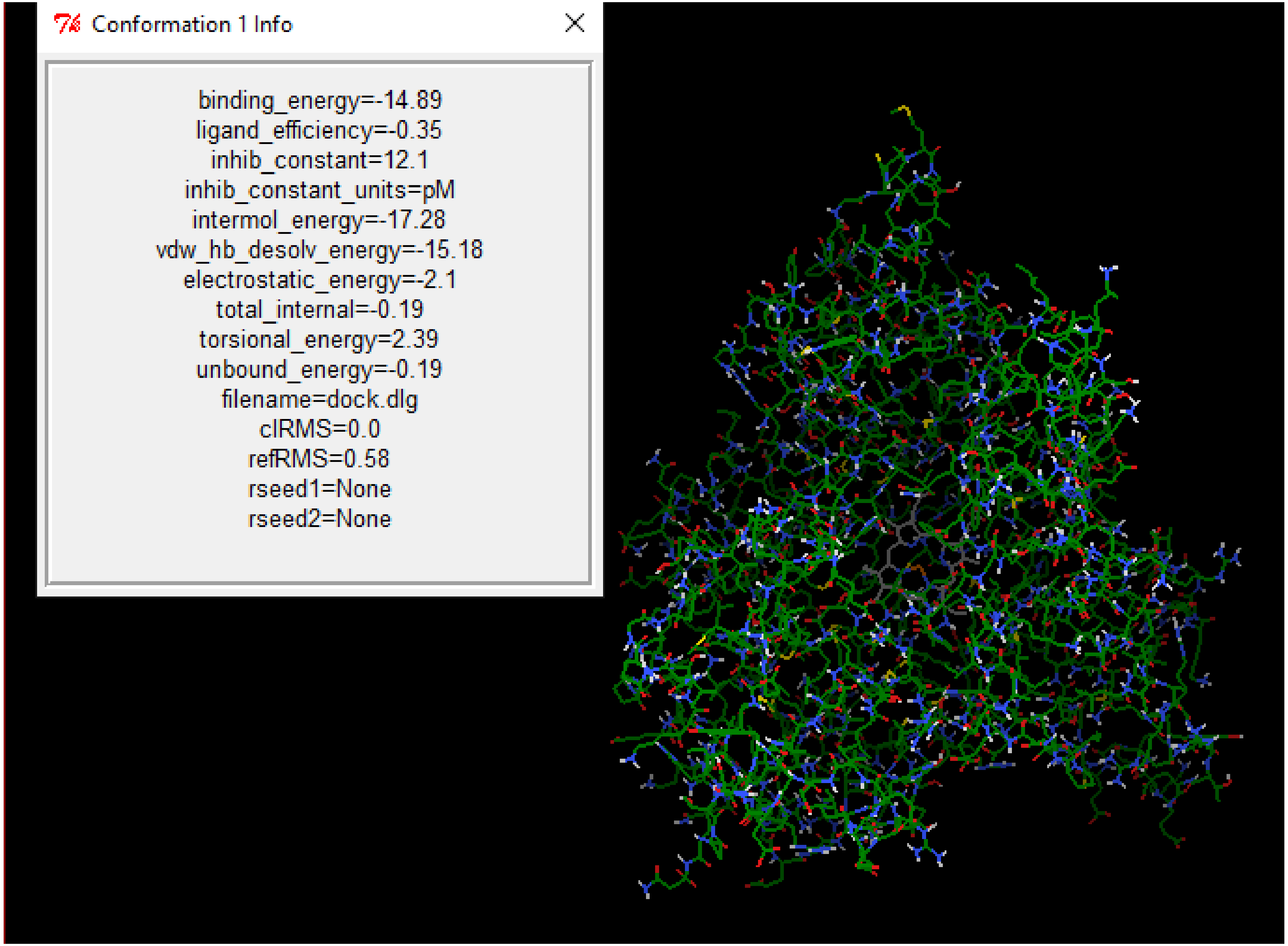

Autodock Result
Mutant

Hydrophobic Interactions ···

Index	Residue	AA	Distance	Ligand Atom	Protein Atom
1	132A	ILE	3.77	3683	683
2	133A	ILE	3.75	3684	690
3	133A	ILE	3.56	3683	692
4	152A	LEU	3.57	3700	858
5	306A	ALA	3.71	3701	2103
6	370A	VAL	3.36	3667	2627
7	430A	PHE	3.61	3671	3130
8	438A	ALA	3.44	3683	3184
9	443A	ALA	3.81	3661	3222

Hydrogen Bonds

Index	Residue	AA	Distance H-A	Distance D-A	Donor Angle	Protein donor?	Side chain	Donor Atom	Acceptor Atom
1	115A	GLN	3.42	3.99	118.34	✔	✔	559 [Nam]	3697 [O.co2]

Plip Protein ligand interaction result Result Mutant

Salt Bridges

Index	Residue	AA	Distance	Protein positive?	Ligand Group	Ligand Atoms
1	145A	ARG	5.04	✓	Carboxylate	3697, 3696
2	375A	ARG	4.33	✓	Carboxylate	3692, 3693
3	435A	ARG	4.16	✓	Carboxylate	3697, 3696

Metal Complexes

Index	Residue	AA	Metal	Target	Distance	Location
Complex 1: Fe, tetrahedral (4)						
1	600A	HEM	3689	3685	2.04	ligand
2	600A	HEM	3689	3686	2.02	ligand
3	600A	HEM	3689	3687	2.01	ligand
4	600A	HEM	3689	3688	2.09	ligand

Plip Protein ligand interaction result Result Mutant

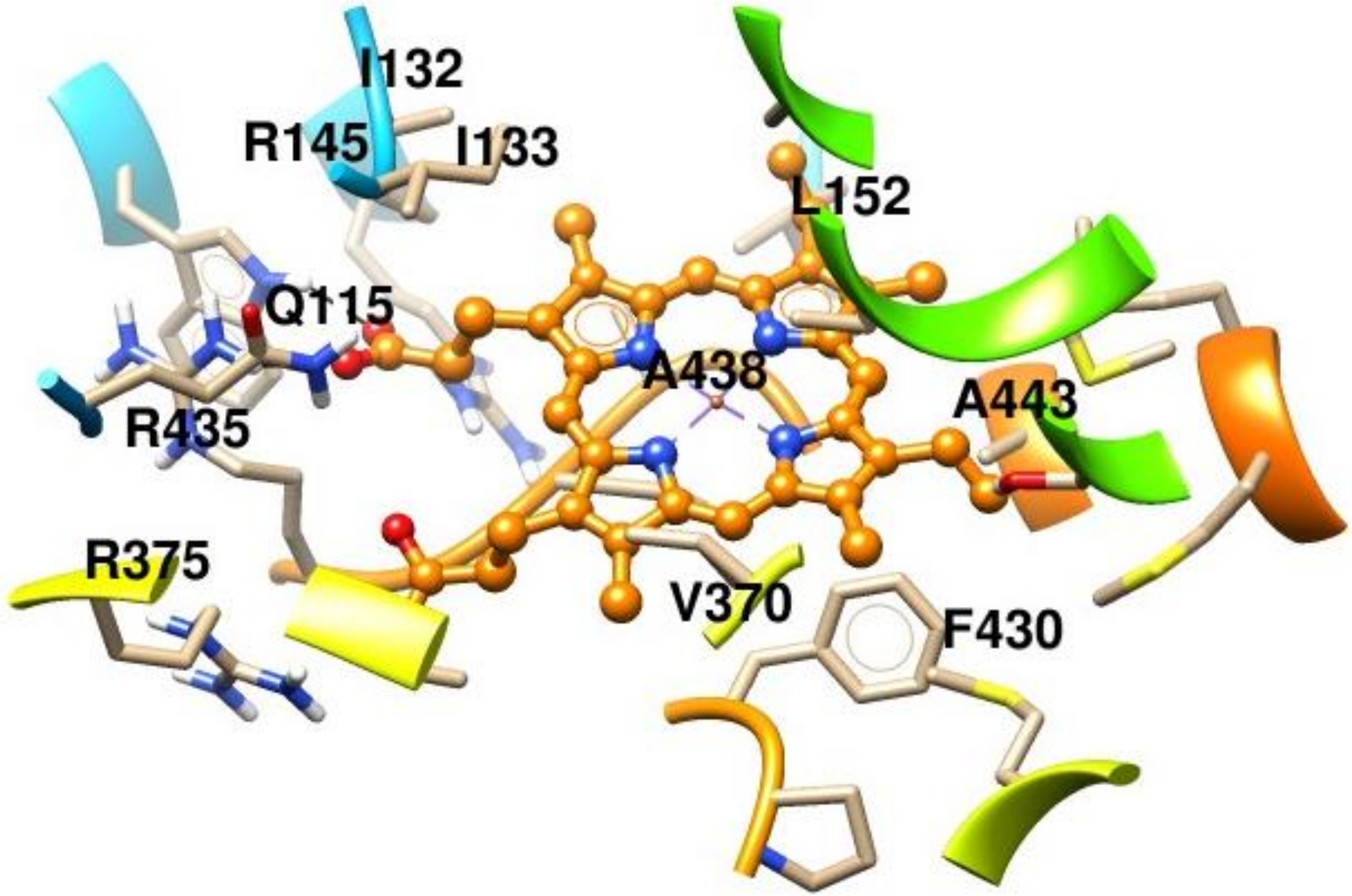

Protein ligand
interaction result
Result Mutant

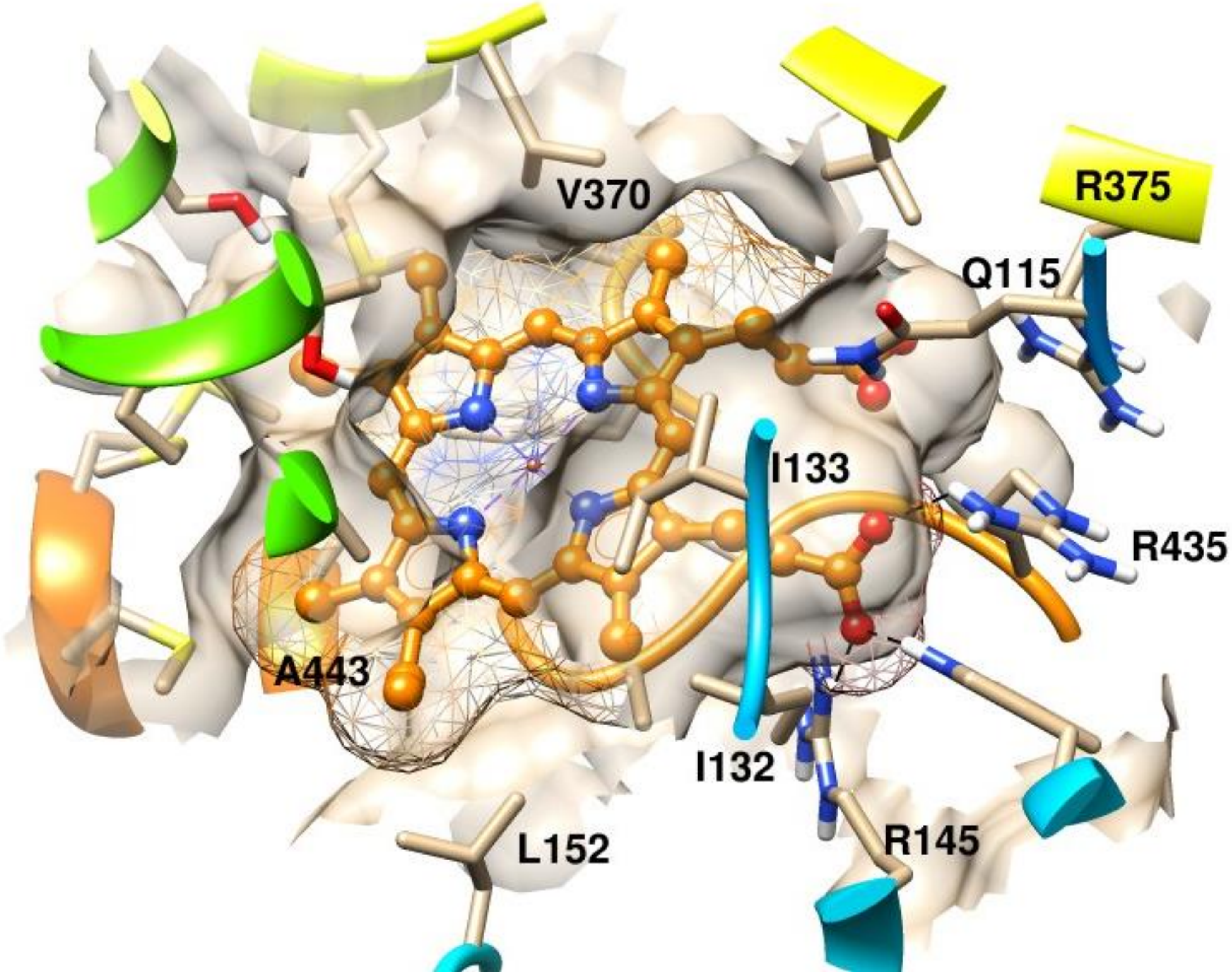

Protein ligand interaction result result Mutant

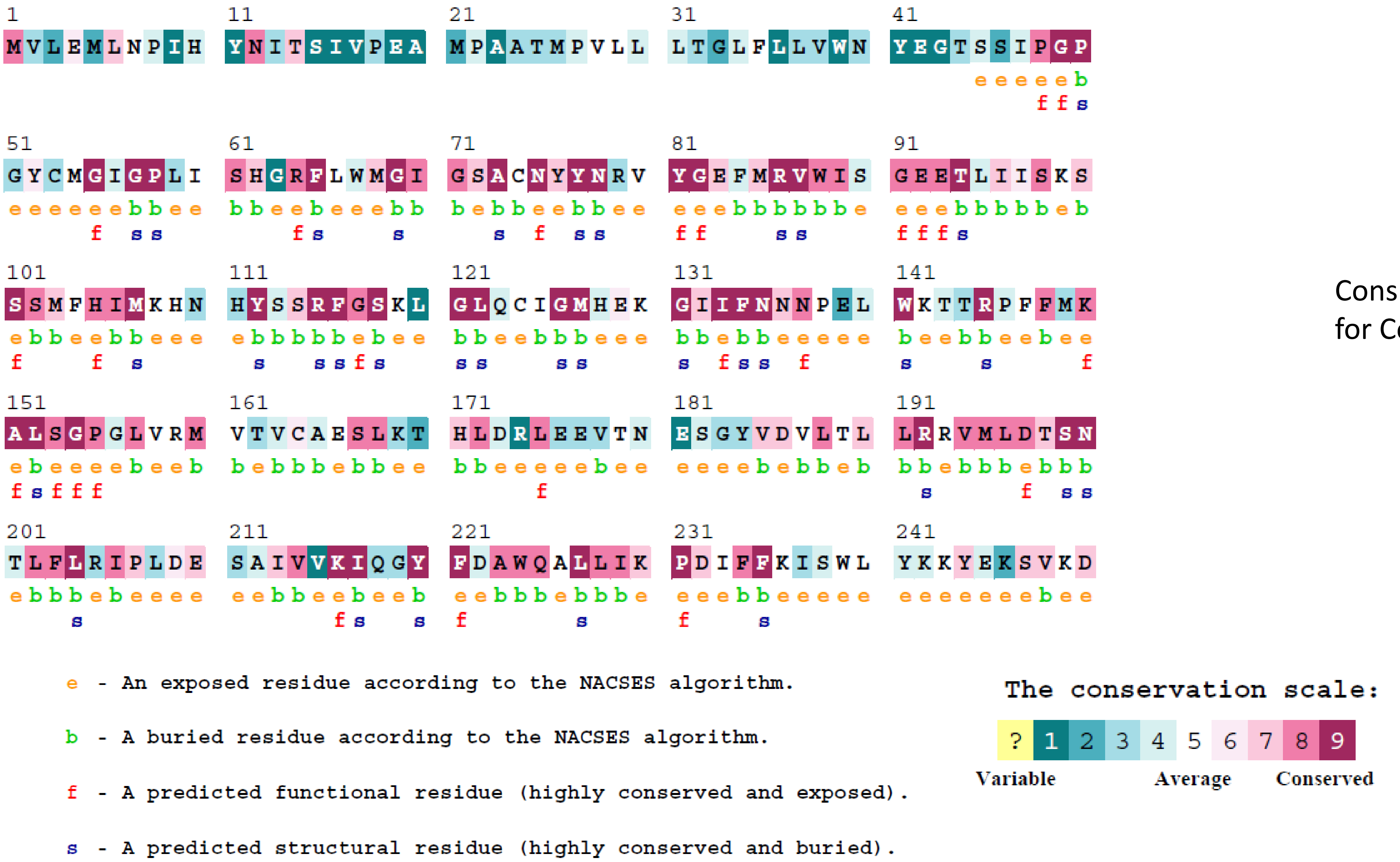

Consurf Analysis for Conservation

Conserved Domain analysis using CDD

Conserved domains on [gi|13904860|ref|NP_112503|]

View Concise Results

aromatase [Homo sapiens]

Graphical summary   Zoom to residue level   show extra options »

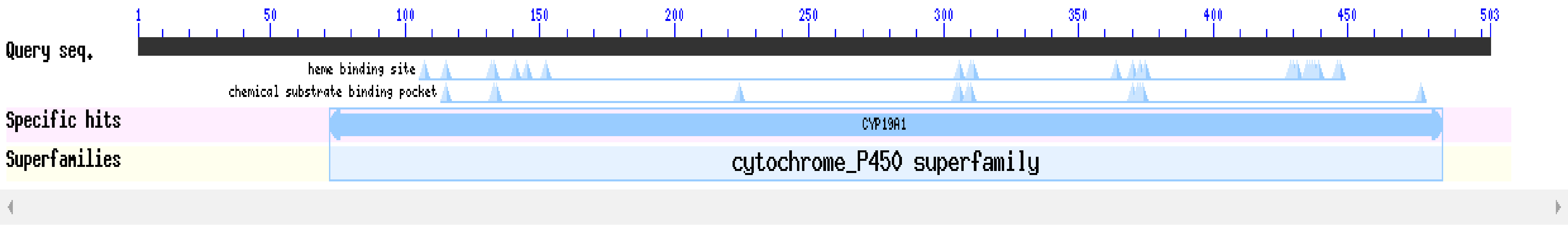

Search for similar domain architectures   Refine search

List of domain hits

Name	Accession	Description	Interval	E-value
[+] CYP19A1	cd20616	cytochrome P450 family 19, subfamily A, polypeptide 1; CYP19A1, also called aromatase or ...	72-485	0e+00

HOPE Server Analysis

The figure below shows the schematic structures of the original (left) and the mutant (right) amino acid. The backbone, which is the same for each amino acid, is colored red. The side chain, unique for each amino acid, is colored black.

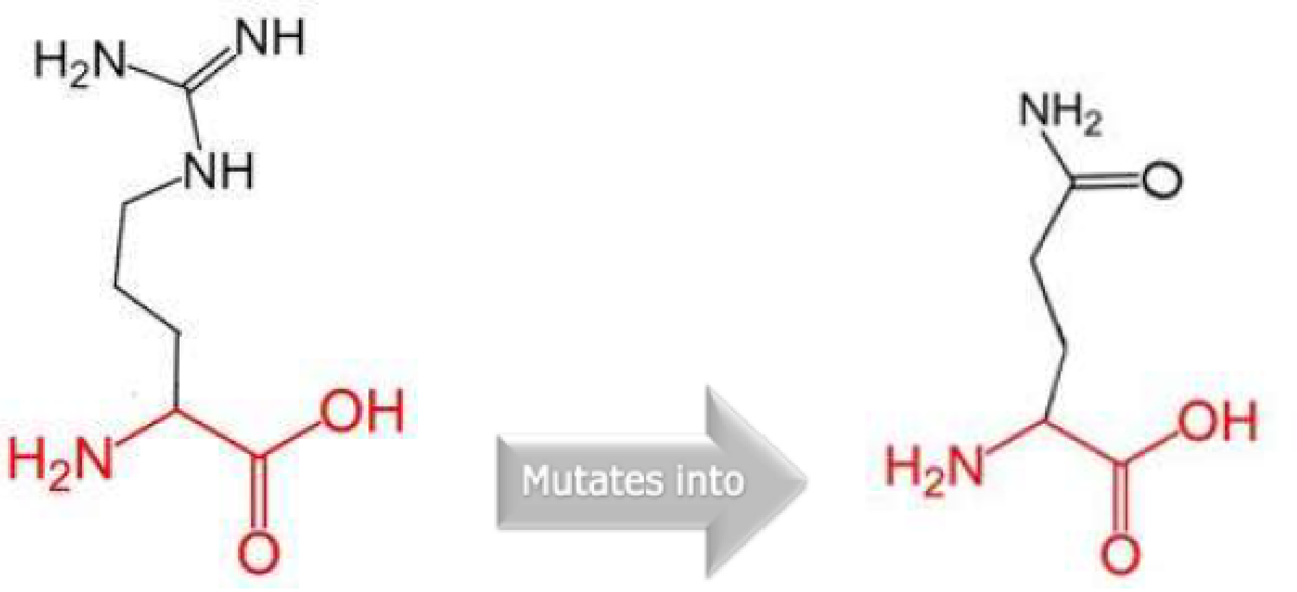

Each amino acid has its own specific size, charge, and hydrophobicity-value. The original wild-type residue and newly introduced mutant residue often differ in these properties.

The mutant residue is smaller than the wild-type residue.

The wild-type residue charge was POSITIVE, the mutant residue charge is NEUTRAL.

The report will evaluate the effect of the mutation on the following features: Contacts made by the mutated residue, structural domains in which the residue is located, modifications on this residue and known variants for

Conservation

Only this residue type was found at this position. Mutation of a 100% conserved residue is usually damaging for the protein.

The mutant residue, however, has some properties in common with the wild-type residue. This mutation might occur in some rare cases, but it's more likely that the mutation is damaging to the protein.

Your mutant residue is located near a highly conserved position.

HOPE Server Analysis

The mutated residue is located in a domain that is important for the activity of the protein and in contact with residues in another domain. It is possible that this interaction is important for the correct function of the protein. The mutation can affect this interaction and as such affect protein function.

The mutated residue is located in a domain that is important for the activity of the protein and in contact with another domain that is also important for the activity. The interaction between these domains could be disturbed by the mutation, which might affect the function of the protein.

The mutated residue is located in a domain that is important for the activity of the protein and in contact with another domain that is known the be involved in binding. The interaction between these domains could be disturbed by the mutation, which might affect the signal transduction between the domains.

Amino Acid Properties

There is a difference in charge between the wild-type and mutant amino acid.

The charge of the buried wild-type residue is lost by this mutation.

The wild-type and mutant amino acids differ in size.

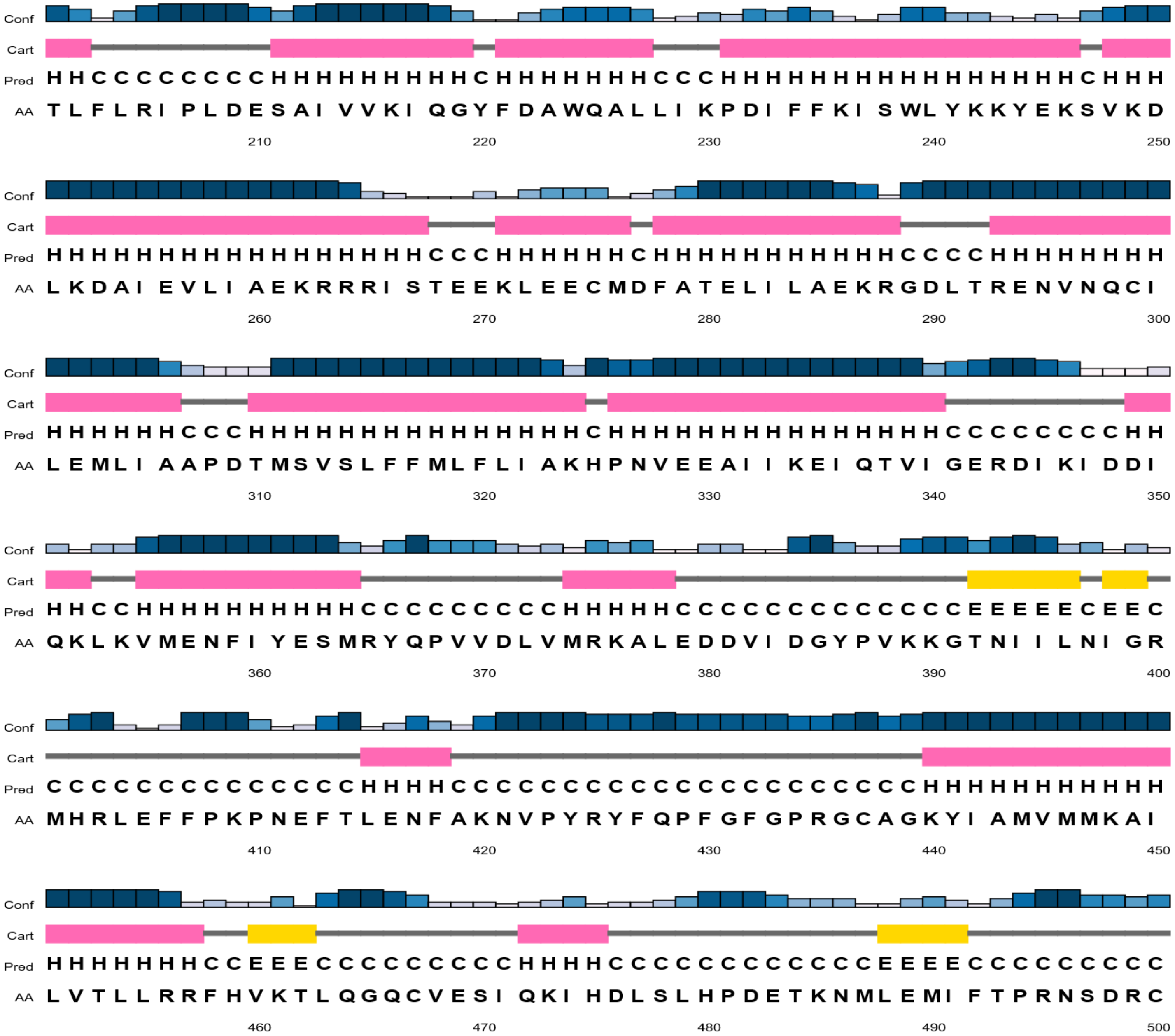

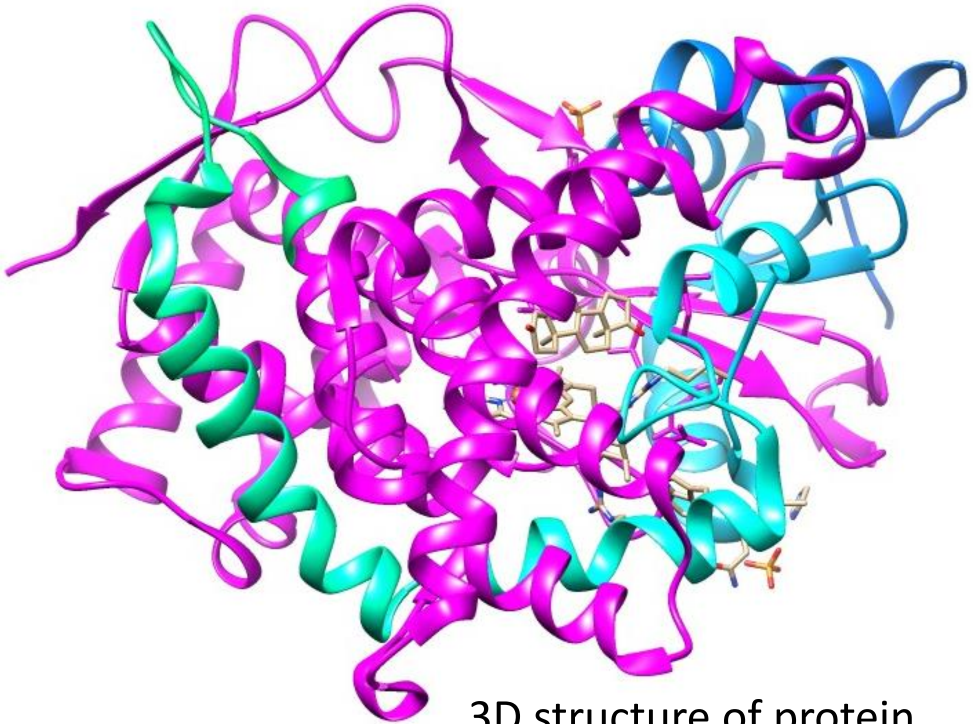

Legend:

- Strand
- Helix
- Coil

3D structure of protein. The truncated protein (loss of tertiary structure) is represented in magenta colour. Left picture shows the Loss of a large chunk of secondary structure elements (PSIPred)
